# Supplementary material for: Histological Profiling of the Human Umbilical Cord: A Potential Alternative Cell Source in Tissue Engineering
Source: J Pers Med. 2022 Apr 18;12(4):648. doi: 10.3390/jpm12040648 (PMC9028794; doi:10.3390/jpm12040648)
Supplement: Supplementary file 1 [file jpm-12-00648-s001.zip › jpm-1600626-supplementary.pdf]

Table S1. Antibodies and reagents used for the immunohistochemistry and immunofluorescence analysis.

Antibody/Reagent	Dilution/Incubation	Pretreatment	Reference
Rabbit Anti-CD31 (PECAM-1)	1:500 Overnight at 4 °C	Citrate buffer pH = 6 20 min at 95 °C	Abcam. Cambridge, UK. (ref. ab76533)
Rabbit Anti-CD34	1:2500 Overnight at 4 °C	Citrate buffer pH = 6 20 min at 95 °C	Abcam. Cambridge, UK. (ref. ab81289)
Rabbit Anti-CD73	1:200 Overnight at 4 °C	Citrate buffer pH = 6 20 min at 95 °C	Abcam. Cambridge, UK. (ref. ab175396)
Rabbit Anti-CD90	1:25 Overnight at 4 °C	Citrate buffer pH = 6 20 min at 95 °C	Abcam. Cambridge, UK. (ref. ab92574)
Rabbit Anti-CD105	1:200 Overnight at 4 °C	Citrate buffer pH = 6 20 min at 95 °C	Abcam. Cambridge, UK. (ref. ab169545)
Rabbit Anti-Collagen III	1:250 Overnight at 4 °C	Citrate buffer pH = 6 20 min at 95 °C	Abcam. Cambridge, UK. (ref. ab7778)
Mouse Anti-Collagen IV	Ready to use Overnight at 4 °C	EDTA buffer pH = 8 20 min at 95 °C Pepsin 10 min at 37 °C	Master Diagnostica. Granada, Spain. MAD-000733QD
Mouse Anti-Fibronectin	1:250 Overnight at 4 °C	EDTA buffer pH = 8 20 min at 95 °C	Invitrogen. Waltham, MA. (ref.11981)
Rabbit Anti-Versican	1:100 Overnight at 4 °C	Chondroitinase 60 min at 37 °C	Abcam. Cambridge, UK. (ref. ab19345)
Mouse Anti-Vimentin	1:200 Overnight at 4 °C	Citrate buffer pH = 6 20 min at 95 °C	Sigma-Aldrich/Merck. Steinheim, Germany. (Cat. no. V6389)
Rabbit Anti-Von Willebrand Factor (vWF)	1:200 Overnight at 4 °C	Citrate buffer pH = 6 20 min at 95 °C	Abcam. Cambridge, UK. (ref. ab6994)
ImmPRESS® HRP Anti-Rabbit IgG (Peroxidase)	Ready to use 30 min at RT	-	Vector Laboratories. Burlingame, CA. (ref. MP-7401)
ImmPRESS® HRP Anti-Mouse IgG (Peroxidase)	Ready to use 30 min at RT	-	Vector Laboratories. Burlingame, CA. (ref. MP-7402)
FITC-conjugated anti-mouse	1:500 1 hour at RT	-	Sigma-Aldrich/Merck. Steinheim, Germany. (Cat. no. F0257)
FITC-conjugated anti-rabbit	1:500 1 hour at RT	-	Sigma-Aldrich/Merck. Steinheim, Germany. (Cat. no. F7367)
